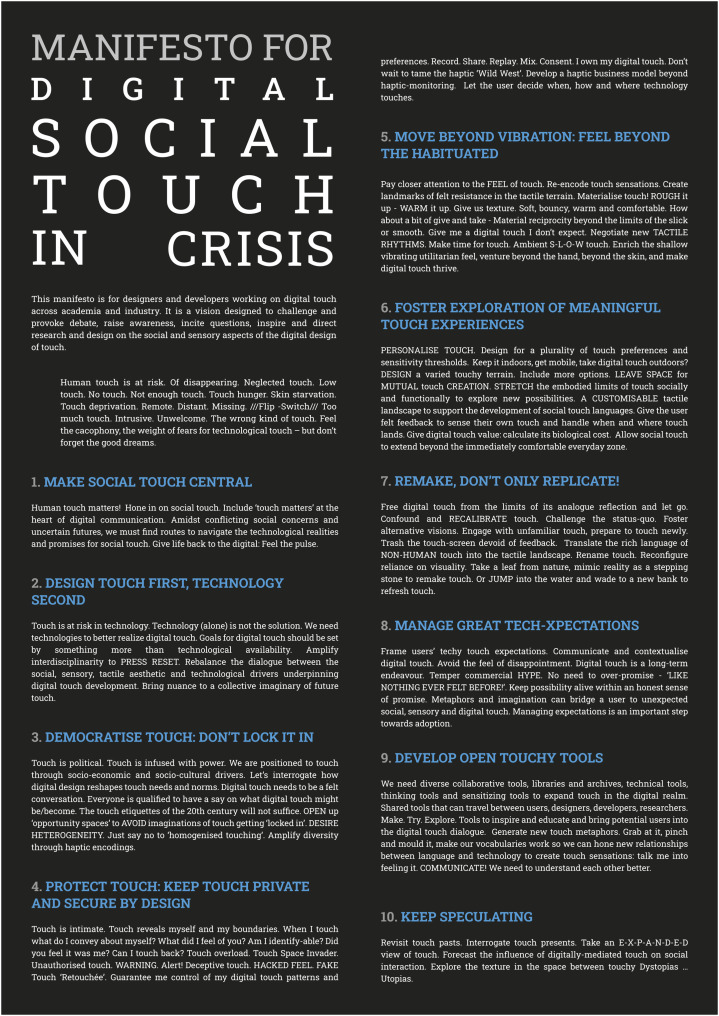# A provocative call to engage with social and sensory aspects of touch

**DOI:** 10.1177/26349795221115463

**Published:** 2022-08-10

**Authors:** Carey Jewitt, Sara Price, Jürgen Steimle, Gijs Huisman, Lili Golmohammadi, Narges Pourjafarian, William Frier, Thomas Howard, Sima Ipakchian Askari, Michela Ornati, Sabrina Paneels, Judith Weda

**Affiliations:** 4919 University College London, UK; Saarland University, Germany; Delft University of Technology, Netherlands; 4919 University College London, UK; Saarland University, Germany; Ultraleap, UK; University of Rennes, France; Vilans, Netherlands; Università della Svizzera Italiana, Switzerland; University Paris-Saclay, France; University of Twente, Netherlands

**Keywords:** Touch, social touch, digital touch, multimodal, sensory, haptics, design, manifesto, interdisciplinary research

## Abstract

The social and sensory aspects of touch are critical for human communication, yet the challenges of haptic technology development and a focus on the technological means that digital touch communication often fails to realise the potential and promise of touch. The *Manifesto for Digital Social Touch in Crisis* responds to this through a call to action to rethink and reimagine digital touch. It offers 10 provocative statements as a resource for how haptic designers, developers and researchers might rethink and reimagine the social and sensory aspects of touch, and foreground these more in design.

This practitioner reflection show the potential of the manifesto form (see Hanna and Ashby, 2022) to bridge between disciplinary boundaries in this case with attention to concerns about social touch. The Manifesto for Digital Social Touch in Crisis was developed through an interdisciplinary collaboration between computer scientists, designers, engineers, Human Computer Interaction scholars and social scientists from industry and academia. The collaboration and manifesto development method are outlined elsewhere (Jewitt et al., 2021).

The manifesto articulates seven foundational themes related to the key opportunities and challenges raised through the growth of digital touch facing designers, developers and researchers. These themes include the need to (1) broaden the conceptualisation of touch; (2) enrich digital touch experience; (3) engage with the wider socio-political context of touch including the commercialisation of digital touch (a theme also articulated in Golmohammadi’s contribution to this special issue); (4) understand and manage user expectations; (5) consider the design of touch privacy; (6) develop interaction design tools; and (7) encourage interdisciplinary dialogue on the social and sensory aspects of touch.

As society engages with and emerges from the uncertainty of touch in Covid-19 times, the *Manifesto for Digital Social Touch in Crisis* signals a desire for change and a rethinking and reimaging of the social and sensory aspects of touch through the design process.